# 2572. Adherence to Evidence-based Guidelines for the Management of Pneumonia in a Tertiary Teaching Hospital in Saudi Arabia

**DOI:** 10.1093/ofid/ofad500.2189

**Published:** 2023-11-27

**Authors:** Abdullah A Alhifany

**Affiliations:** Umm Al-Qura University, Makkah, Makkah, Saudi Arabia

## Abstract

**Background:**

Abstract:

Background

Adherence to therapeutic guidelines is crucial when treating pneumonia, as it reduces mortality rate, length of hospital stay and duration of antibiotic therapy. However, the high non-adherence rate to treatment guidelines, in general, and Infectious Disease Society of America (IDSA) guidelines, are still reported globally. According to our knowledge, no existing data is available regarding the rate of physicians' adherence to the IDSA guidelines for managing pneumonia in Saudi Arabia. Therefore, we aim to assess the adherence rate and the clinical outcomes among patients treated according to the IDSA guidelines, in a tertiary care center in Riyadh.

**Methods:**

Methods

A single-centered, retrospective observational study was conducted at King Khalid University Hospital, Riyadh, Saudi Arabia. All data were extracted from the hospital's electronic information system, known as Esihi. Adult patients (≥18 years old) who have been diagnosed and treated in the hospital for community-acquired pneumonia, hospital-acquired pneumonia, or ventilator-associated pneumonia from Nov 2019 to Nov 2021 were included.

**Results:**

A total of 148 patients were included in this study, with an average age of 65 ± 20 years. Demographics and baseline characteristics of the included patients are shown in Table 1. The data showed that only 74 (50%) were treated according to the guidelines' recommendations, while the other 74 (50%) were not. The former group had a statistically higher cure rate and lower mortality compared to the latter (95% vs. 84%; p = 0.034) and (5% vs. 14%; p = 0.022), respectively (Figure 1). Nevertheless, patients who were treated according to the guidelines had a higher CURB-64 score for pneumonia severity and Average Calculated Charlson comorbidity index than patients who were treated irrespectively of the guidelines (1.86 vs 1.39) and (4.62 vs 3.28), respectively, yet, no statistically significant difference was observed (Table 1 and 2).

**Figure:**
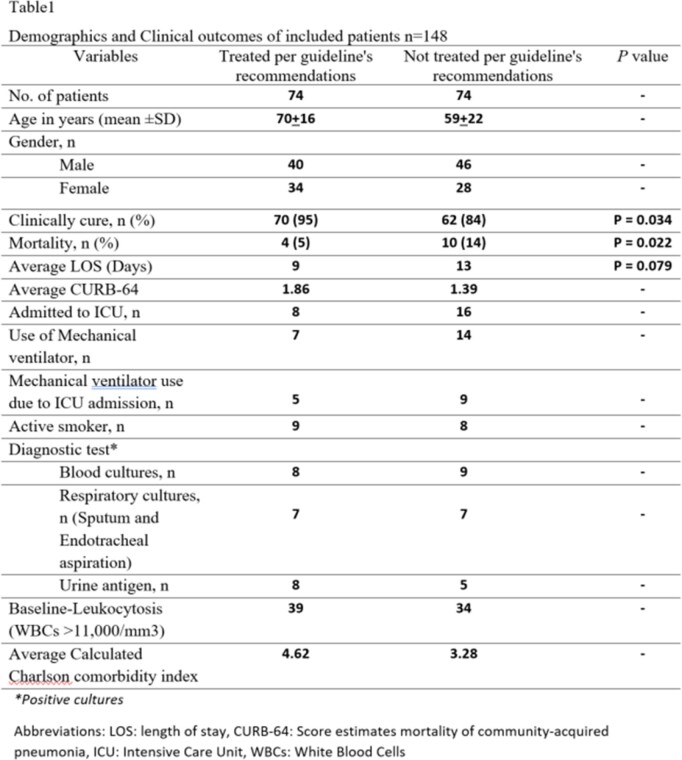

**Figure:**
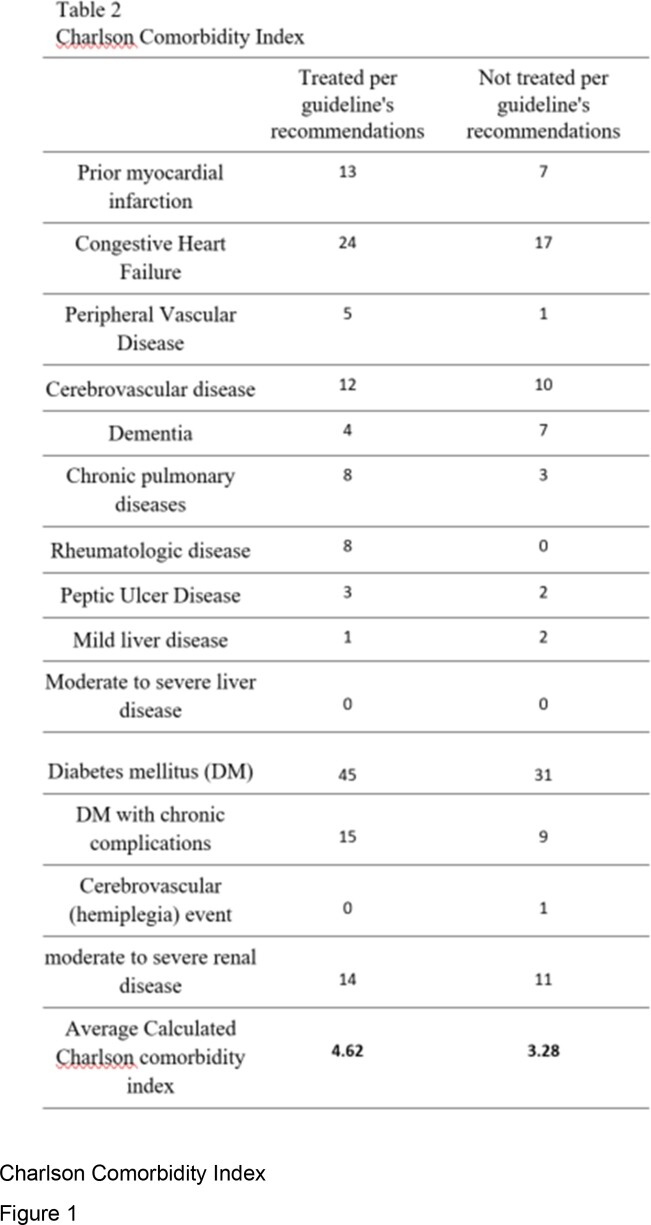

**Figure:**
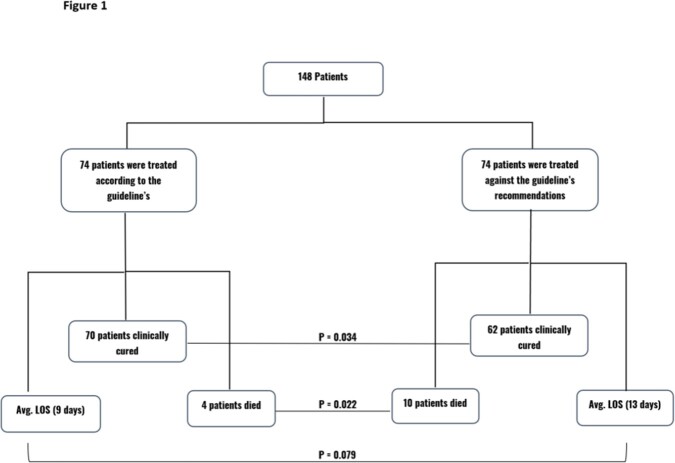

Demographics and Clinical outcomes of included patients n=148

**Conclusion:**

Conclusion

Non-adherence to the evidence-based guidelines has been observed in 50% of patients treated for pneumonia. In addition, higher clinically cure rate, shorter length of stay and lower mortality rate have been observed in patients who were treated based on the evidence-based guidelines.

**Disclosures:**

**All Authors**: No reported disclosures

